# Harnessing the Power of Microbiome Assessment Tools as Part of Neuroprotective Nutrition and Lifestyle Medicine Interventions

**DOI:** 10.3390/microorganisms6020035

**Published:** 2018-04-25

**Authors:** Miguel Toribio-Mateas

**Affiliations:** School of Health and Education, Middlesex University, The Burroughs, London NW4 4BT, UK; mt928@live.mdx.ac.uk

**Keywords:** microbiome, neuroprotection, microbiota-gut-brain axis, precision nutrition, real-world data, lifestyle medicine

## Abstract

An extensive body of evidence documents the importance of the gut microbiome both in health and in a variety of human diseases. Cell and animal studies describing this relationship abound, whilst clinical studies exploring the associations between changes in gut microbiota and the corresponding metabolites with neurodegeneration in the human brain have only begun to emerge more recently. Further, the findings of such studies are often difficult to translate into simple clinical applications that result in measurable health outcomes. The purpose of this paper is to appraise the literature on a select set of faecal biomarkers from a clinician’s perspective. This practical review aims to examine key physiological processes that influence both gastrointestinal, as well as brain health, and to discuss how tools such as the characterisation of commensal bacteria, the identification of potential opportunistic, pathogenic and parasitic organisms and the quantification of gut microbiome biomarkers and metabolites can help inform clinical decisions of nutrition and lifestyle medicine practitioners.

## 1. Introducing the Microbiota-Gut-Brain Axis

A growing body of preclinical and clinical evidence supports the concept of bidirectional microbiota-gut-brain interactions. The complexity of this intricate communication system is such, and the amount of studies published every week is so large, that this paper only attempts to provide precision-oriented practitioners seeing patients with gut health and cognitive impairment issues with an overview of the subject, focusing only on salient aspects in current literature that have clear implications for clinical practice.

The microbe population living in our gastrointestinal (GI) tract, collectively known as “gut microbiota” (and traditionally referred to as “gut flora”), interacts with the human host through immune, neuroendocrine and neural pathways. Bacteroidetes and Firmicutes are the two main phyla in the GI tract [1] with quantity and diversity increasing from stomach to small intestine to colon [2,3]. The human microbiota is able to cast local, as well as systemic effects on host biology, both in health and disease. For example, gastrointestinal dysbiosis (an alteration in normal commensal gut microbiota with an increase in pathogenic microbes, which deranges homeostasis) has been consistently reported as a key contributory factor to the development of metabolic disease [4,5,6]. Consumption of ultra-processed foods has long been considered a factor contributing to dysbiosis. The NOVA food classification [7] considers most hyperpalatable, highly-processed foods typically seen in “Western diets”, including carbonated drinks, margarines and spreads, cookies, biscuits, breakfast cereals, energy bars, energy drinks, prepared pies, pizzas, meat nuggets and pre-packaged or “ready meals” as ultra-processed foods, and associations between consumption of ultra-processed foods and conditions such as cancer have been reported by researchers in recently published large-scale population based studies such as the NutriNet-Santé prospective cohort study [8].

Clinicians who are only starting to realise how important the relationship between gut dysbiosis and systemic disease is may find it beneficial to do some preliminary reading on this subject. The reviews by Bibbo et al. [9], Milani et al. [10], Ojeda et al. [11] and Conlon and Bird [12] illustrate this relationship beautifully.

More specifically, as examples of how dietary patterns have the ability to contribute to dysbiosis, the reader may find that the following papers highlight the effects of a “Western diet” on the gut microbiota and how changes mediated by a diet that is typically low in fibre from fresh foods, but high in refined carbohydrates, and damaged dietary fats from ultra-processed foods are seen as instrumental in the emergence of symptoms relating to metabolic disease, including cardiovascular [13,14], obesity [15], as well as other related conditions like asthma [16,17,18,19] and oestrogen dysregulation [20,21], to name but a few.

### Dysbiosis and Leaky Gut

Poor dietary fibre is only one of many environmental influences associated with the increase in chronic inflammatory disorders. Karkman et al. [22] argue that a lack of contact with natural biodiversity along with excessive antibiotic use are also key modulators of microbial diversity. Additionally, Ananthakrishnan et al. [23] and Salim et al. [24] agree that the progressive contamination of the environment by countless toxic compounds contributes to gut dysbiosis and acts as a trigger for deranged host immune responses and mucosal defences.

Microbiologists know that the gut microbiota is capable of regulating host fat deposition, metabolism and immune function and that other environmental influences such as exercise [25,26,27] or sleep [28,29] are important factors in determining changes in microbial composition that impact the composition of the microbiota. For example, a recent study by Karl et al. [30] found that individuals that engaged in intense physical activity experienced increased α-diversity and relative abundance in more than 50% of bacterial genera identified by 16S rRNA (ribosomal ribonucleic acid) sequencing. This is a particularly interesting case that illustrates the positive effects of exercise on both diversity and abundance. However, the study also found that the intense physiological stress brought about by a four-day cross-country ski-march increased intestinal permeability or “leaky gut”, as confirmed by quantification of urinary excretion of orally-ingested sucralose and mannitol. The author questions whether the effects of exercise on intestinal permeability are hormetic, i.e., whether there is indeed a threshold past which the benefits of physical activity quickly turn into detriment. In any event, the microbial ecosystem is very complex and so is the host-bacteria interaction. Future research will hopefully enable researchers to answer this and other puzzling questions.

It is also known that microbial diversity is highly plastic early in life, as highlighted by a recent study by Berding et al. [31], which found that both bacterial composition and short chain fatty acid concentration in children aged 4–8 years is distinctly based on specific dietary patterns. We also know that postnatal maturation of immune regulation is largely driven by infant exposure to microbes, as confirmed by recent clinical evidence published by Xiao et al. [32]. In their study of 200 exclusively formula-fed healthy babies aged 4–6 months, the researchers found that infants who were administered a supplement containing *Bifidobacterium infantis* R0033, *Bifidobacterium bifidum* R0071 and *Lactobacillus helveticus* R0052 for two weeks experienced a beneficial increase in secretory IgA compared with those receiving an identical placebo. However, looking into the microbiota composition of 300 healthy Danish babies, Bergström et al. [33] found that that the effects of breastfeeding on the microbiota are no longer prevalent after 36 months. In fact, the study concluded that there was actually no difference between bottle feeding or breast feeding past the 36-month point. Additionally, looking at even-earlier-life modulation, evidence of in utero programming of the immune system is mounting. For example, in a double-blind, randomised, placebo-controlled trial, Rutten et al. [34] documented the experience of pregnant mothers who took a supplement containing a probiotic mixture consisting of *B. bifidum* W23, *B. lactis* W52 and *Lc. lactis* W58 for the last six weeks of pregnancy. The same supplement was administered to their offspring during their first year of life, and faecal samples were analysed over a six-year period. The probiotic strains used in the intervention were found in higher abundance and prevalence in the probiotic group during supplementation, but the changes were not found to be long-lasting.

Despite the plasticity of the gut microbiome during early life, exposure to factors that affect microbial diversity during childhood have been posited to have consequences much later in life. For example, in a study of 139 children of between seven and 15 years of age living in Mexico City, Calderon-Garciduenas et al. [35] found that those with the highest levels of pollutants in cerebrospinal fluid also had the highest levels of zonulin, a protein discussed in this paper as a useful tool to assess endothelial tight junction integrity, a factor that the researchers saw as a contributor to “the neuroinflammatory pathology hallmarks of Alzheimer’s and Parkinson’s, and present in Mexico City children”.

Aside of poor dietary fibre intake, other factors known to contribute to increased intestinal permeability include nutritional deficiencies, e.g., vitamin A [36], vitamin D [37], zinc [38], magnesium [39] and, based on animal models, a high-fat/high-carbohydrate diet [40] and a high-fructose diet, both of which have the potential to induce changes in gut microbiota leading to microbial dysbiosis, metabolic endotoxemia and inflammation that could contribute to increased intestinal permeability, traditionally referred to as “leaky gut”, seen alongside increased blood-brain barrier permeability mediating the pathogenesis of neurodevelopmental disorders such as autism [41], neuroimmune dysregulation disorders such as multiple sclerosis [42,43,44] and neurodegenerative conditions such as Alzheimer’s disease (AD) [45,46]. A disrupted intestinal barrier is also seen in cognitive dysfunction, as well as in anxiety and depression [47,48,49]. In fact, although the primary risk factor for AD is advancing age, other factors such as hyperinsulinaemia/diabetes mellitus type 2 (DMT2), hyperlipidaemia, obesity, vascular factors and depression play a role in its pathogenesis [50,51,52]. Indeed, the specific role of gut microbiota in modulating neuroimmune functions well beyond the gastrointestinal tract may influence the development of neurodegenerative processes. As an example, alpha-synuclein deposition and the associated neurodegeneration that takes place in the enteric nervous system contribute to gastrointestinal dysfunction [53], increased intestinal permeability [54], increased oxidative stress [55] and local inflammation [56] that feature alongside constipation in Parkinson’s disease (PD) patients.

## 2. The Microbiota-Gut-Brain Axis: A Complex Communication System

Emerging data from both animal and human studies suggests that the function and health of the central nervous system, and the brain as the principal organ in it, is modulated by the complex interaction amongst a number of factors. Multiple routes of communication between the gut and brain have been established, and these include the vagus nerve (VN), the immune system, short chain fatty acids and tryptophan [57]. It is worth highlighting that the vagus nerve is considered by some authors to be the principal interface of the microbiota-gut-brain axis [58,59]. This assumption is based on animal studies by Agostini et al. [60] that described how the VN comprises up to 80% afferent and only around 20% efferent fibres, respectively. These early neurogastroenterology anatomical explorations make a lot of sense to current researchers. Indeed, according to Sundman et al. [61], the channels of communication between the gut and the brain encompass and are influenced by the following factors:

(1) the composition/diversity of the gut microbiota;
(2) neurotransmitters, hormones, and immune- and neuro-peptides produced in the gut and communication between these and gut microbes;
(3) the integrity of the intestinal wall serving as the physical barrier to the external environment.

The influence of the gut microbiota on the function of the central nervous system (CNS) is manifested in both normal and disease conditions, and it is seen from early age, as documented in a recent study on 77 toddlers ranging from 18–27 months of age where researchers found differences in gut microbiome composition, including alpha and beta diversity plus relative abundances of specific bacterial species, in association with children’s temperament [62].

In disease states, the basic mechanism of action is mediated by inflammation triggered by loss of the natural eubiotic or balanced state of the gastroinstestinal (GI) tract and its progression towards loss of homeostasis, i.e., dysbiosis [63,64]. For example, the low-grade, often chronic inflammation and/or immune activation that underlies the aetiology of irritable bowel syndrome (IBS) is seen as an increased risk factor in mood disorders such as depression [65,66,67], anorexia nervosa [68], obsessive compulsive disorder (OCD) [69] and autism [70]. Additionally, this type of abnormality in the gut-brain axis affecting individuals with IBS and IBS-like symptoms is seen to be associated with several chronic non-communicable disorders including, but not limited to, chronic fatigue syndrome (CFS)/fibromyalgia [71,72], obesity [73,74], cardiovascular disease [75,76], DMT2 [77,78] and psoriatic arthritis [79]. Further, in inflammatory bowel disease (IBD, e.g., Crohn’s disease and ulcerative colitis), patients’ perception of their own health is also impaired, which has been shown to have a negative impact on self-reporting of health-related quality of life [80]. For example, based on a cross-sectional study of 147 IBD patients, Freitas et al. [81] reported that anxiety-triggered symptoms are the most potent independent correlate of most aspects of health-related quality of life.

The microbiota-gut-brain axis involves a number of sophisticated channels of communication amongst many interconnected systems, including the CNS, the autonomic nervous system (ANS), the HPA axis, often referred by laypeople as “the stress system”, as well as the GI corticotropin-releasing factor system, and the intestinal immune response system featuring the intestinal mucosal barrier and the luminal microbiota [58,82,83]. Taking all of these components into account, it is easy to appreciate how regulation of the CNS by the gut microbiota is achieved not only through neural, but also through endocrinal, metabolic and immunological pathways.

Neural communication pathways lay within the enteric nervous system (ENS), a main division of the ANS that governs GI function and vagal afferent nerves (VAN) that transmit sensory information from the visceral organs to the CNS. Receptors expressed on VAN are able to sense regulatory gut peptides such as leptin and ghrelin [84], as well as information contained in nutrients such as carbohydrates or fat [85,86,87], relaying these signals to the CNS [88,89].

**Figure 1:**
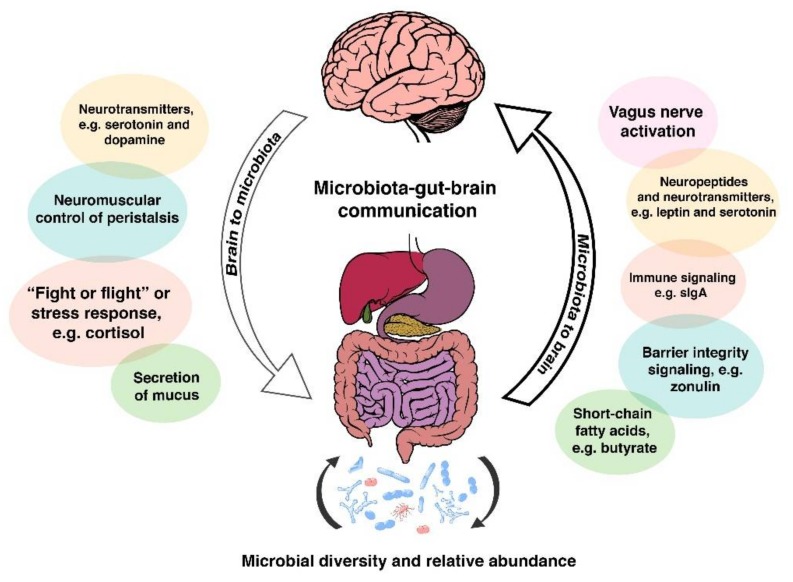
A simplified diagrammatic illustration of the bidirectional communication pathways between the gut microbiota and the brain, featuring a range of molecules originating in the gut that are involved in the upstream part of the communication system. Also featured is the “fight or flight” response, driven by the activation of the sympathetic nervous system, and how exposure of to that response plays an important role in the dysregulation of the intestinal ecosystem.

The microbiota-gut-brain axis also has the ability to alter intestinal permeability and motility through the release of mucus rich in immune molecules such as secretory IgA (sIgA) and neurotransmitters, e.g., serotonin, melatonin, gamma-aminobutyric acid, histamines and acetylcholine [90,91]. Gut microbiota also synthesise nutrients that are essential for optimum human health span. As an example of the metabolic activity of some human gut bacteria, some vitamin B12 (cobalamin) is produced by *Lactobacillus reuteri* [92] and *Lactobacillus plantarum* [93]. Vitamin B12 is important for the development of the nervous system during the early years [94], as well as for healthy brain ageing later in life [95]. Additionally, reduced B12 levels have been seen in autism and schizophrenia [96]. Some of these pathways are summarised in Figure 1 below.

Despite the mounting evidence of the contribution of enteric microbiota to the gut-brain axis in animal models, the study of the complex signalling pathways involved is still in its infancy [97,98]. It is therefore prudent for practitioners to read through the current scientific evidence and to always err on the side of caution when translating the theory into clinical applications for human health.

### From Eubiosis to Dysbiosis and Back

Consumption of a diet high in a combination of fats and refined carbohydrates, and particularly sugar, has the ability to effect long-lasting changes in the healthy microbiota composition, leading to an imbalanced microbial population [99,100]. Overgrowth of pathogenic bacteria results in an increase in lipopolysaccharide (LPS) levels, which triggers the production of pro-inflammatory cytokines in the gut [101,102]. For example, in irritable bowel syndrome (IBS), alterations in microbial diversity [103] are seen in addition to gut-related inflammation as pivotal mediators of endotoxemia, systemic inflammation and neuroinflammation, all of which are documented as contributing factors to cognitive impairment [104,105,106,107,108,109]. Many of the foods that abound in Western diets are rich in substances such as gliadins (a component in gluten), which have endotoxin-like effects [110] that can elicit aberrant immune responses [111,112]. Other proteins such as casein and zein have also been observed to have similar effects in animal models [113].

Nutrition and lifestyle medicine practitioners are only too aware of how psychosocial stress can affect gut function [60,114,115]. This stress normally manifests itself as changes in bowel motion regularity and/or stool consistency [116,117], most likely triggered by secondary changes in intestinal microbiota composition [118,119]. In animal studies, long-term stress has been seen to trigger low-grade inflammation that disturbs gut microbiota, mediating epithelial abnormalities and altering bacterial-host interactions [120]. Furthermore, from research in mice, we know that the presence or absence of exposure to certain microorganisms contributes to individual differences in stress vulnerability [121].

Key questions that emerge out of reviewing the current literature on this subject include whether dysbiosis can be reversed, and whether this reversal might have a measurable neuroprotective effect, e.g., improved cognitive performance. Practitioners with a whole-person clinical approach who have been using nutrition and lifestyle “prescriptions” to help restore a more eubiotic intestinal environment in their patients’ guts will be glad to know that such prescriptions are starting to be backed up by clinical evidence. For example, a recent small-scale human study tested the effect of a probiotic (*B. longum* 1714 strain) on stress response in 22 healthy males and found that the intervention group benefited from improved memory and reduced stress compared to controls [122]. In another human study, this time an all-female, double-blind randomised controlled trial (*n* = 60), participants who received a three-week course of a multi-strain probiotic described by the researchers as a “psychobiotic” reported improved psychological wellbeing, as well as significant differences body composition, compared to controls [123]. These findings are encouraging and provide practitioners with evidence that they may use to support their clinical decisions. 

## 3. Assessing Function by Means of Laboratory Testing and “*n* = 1 Trials”

We share one third of our gut microbiota with people around us, while two thirds are specific to each one of us. This means that the peculiarities in our gut microbiota can provide both scientists and clinicians with information about our health and susceptibility to the development of various diseases. Research on the gut microbiome has boomed in the last 5 years, with 4364 papers published on PubMed in 2017 alone, compared with just 865 in 2012 (PubMed search string: *gut microbiome or gut microbiota*). This explosion of interest in gut microbes has been partly brought about by the increased availability of laboratory tests that enable scientists to analyse microbial genes using a small stool sample to map out thousands of different gut microbes [124]. Thanks to this latest generation sequencing based on 16S technology, we know that in a human stool sample we are likely to find tens of trillions of microorganisms, including at least 1000 different species of known bacteria with more than three million genes. Apart from this number being substantially higher to that of our own human genes, an estimated 150–400-fold more genes than the human genome [125,126], the advantage of stool testing over genetic profiling is that a single stool test can provide far more directly actionable findings about a person’s health than a genome screen, particularly if test results provide data on metabolites such as short chain fatty acids in addition to microbial identification and quantification.

Microbial sequencing and basic metabolomics are easy to translate into powerful clinical applications by practitioners who can act upon any deranged markers identified using a prescription that is almost entirely food-based. In some cases appropriately selected nutraceuticals for which there is good quality evidence of effectiveness can add an edge to the effectiveness of the treatment plan. Simple lifestyle changes such as increased physical activity, improved stress management, or better sleep modulate the activity of the hypothalamic-pituitary-adrenal (HPA) axis and are also known to have an impact on the health of the human microbiome [127].

## 4. Helpful Biomarkers in Stool Tests

From a clinician’s perspective, a selection of helpful biomarkers for practitioners to assess gut-brain function is included below. Please be mindful of the fact that this is not an exhaustive list and that the author’s focus is on the translatability of these markers into nutrition and lifestyle medicine prescriptions.

### 4.1. Microbial Diversity

It appears that individuals suffering from ill-health tend to show narrowing in microbial diversity and that this renders them more susceptible to infection and consequently negatively affects their innate immune function. As an example, a recent prospective observational cohort study analysed the faecal microbiota of 34 patients admitted to an intensive care unit. A significant decrease in bacterial diversity was observed in 50% of the patients, with 13 of the 34 patients having one single bacterial genus making up more than 50% of their total gut microbiota composition. In four out of the 34 patients, a single genus made up 75% of their gut microbiota [128]. Similar evidence is also available for critically ill children [129].

Ageing also seems to affect microbial diversity. Maffei et al. [130] documented in 2017 that biological age, but not chronological age, correlates with a decrease in stool microbial diversity. Similarly, based on data emerging from studies on the effects of faecal microbiota transplantation (FMT), we are now beginning to discover that pathological ageing is associated with a narrowing in microbial diversity, whilst healthy ageing correlates with a more diverse microbiota [131]. In fact, from the data analysis of 728 female twins in the Twins UK database, we also know that there is a strong negative association between frailty and gut microbiota diversity, underpinned by specific taxonomic associations [132]. And in PD, emerging human evidence points to deranged oral and nasal microbial ecosystems [133] and not just to differences in gastrointestinal microbial diversity [134] as factors contributing to the pathogenesis of the condition.

Intestinal microbial dysbiosis with low microbial diversity is also seen in a variety of psychiatric conditions, including eating disorders. As an example, recent human evidence points to gut dysbiosis and aberrant diversity as underlying factors in anorexia nervosa (AN) [135,136,137].

#### 4.1.1. Getting “a Mediterranean Gut”?

As discussed previously in this paper, diets that are rich in ultra-processed foods and poor in micronutrients, like the typical Western or Standard British and American Diets, are associated with higher incidence of conditions such as obesity, cancer and decline in cognitive function. On the other hand, based on a randomised clinical trial parallel to the original PREDIMED study, published in 2013, we know that a Mediterranean-style diet supplemented with olive oil or nuts is associated with improved cognitive function and that these improvements are significant compared with those experienced by participants in the control group who were fed a low-fat diet instead [138]. Additionally, random subsample analysis of the PREDIMED study confirmed that participants allocated to an extra-virgin olive oil-rich diet experienced less cognitive impairment than controls [139]. Bioactive substances such as polyphenols, plant compounds abundant in the Mediterranean dietary pattern, have been associated with improved microbial diversity that correlates with improved mood [140], cognition and cardiovascular measures [141], as well as enhanced blood flow to the brain [142,143], among other benefits.

**Figure 2:**
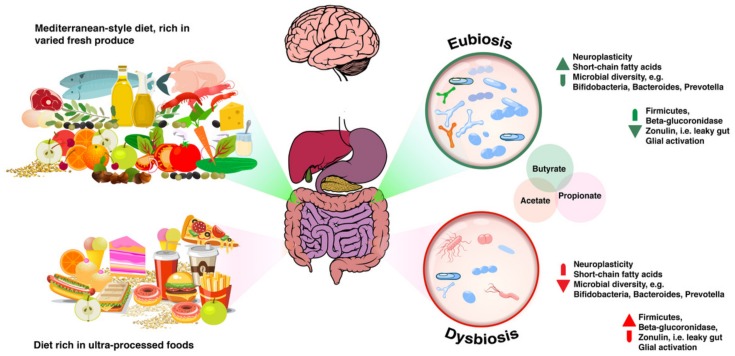
Dietary patterns that are rich in fresh produce, including a variety of brightly-coloured vegetables and fruits, olive oil, nuts and seeds, e.g., a Mediterranean-style diet, are seen to promote eubiosis, contributing to higher levels of all three short chain fatty acids and to a wider microbial diversity, with lower relative abundance in Firmicutes. Other characteristics of a eubiotic gut ecosystem include lower levels of beta-glucuronidase, documented to help with normal elimination of toxicants, and lower zonulin levels, seen as an indication of reduced susceptibility of damage to the intestinal barrier, i.e., less “leaky gut”. In this scenario, less glial activation is observed, resulting in decreased oxidative stress and increased neuroplasticity. On the other hand, dietary patterns rich in ultra-processed foods, and particularly those rich in refined carbohydrates combined with high fat levels are seen to promote gut dysbiosis. Lower microbial diversity, as well as lower levels of short chain fatty acid levels are seen in patients whose diets consist of mostly of ultra-processed foods, with higher relative abundance of Firmicutes. Other markers are also affected. Beta-glucuronidase may be higher, which could pose issues with toxicant elimination via reduced activity of phase II detoxification pathways. There is also a higher susceptibility for barrier tissue damage. Higher zonulin levels in stool would give practitioners an indication that this is the case. A disrupted intestinal barrier tends to be correlated with increased free radical damage to brain tissue, thereby increasing the chances of neurodegeneration, as well as anxiety, depression and other psychiatric disorders.

Based on emerging evidence, the author argues that these food-derived bioactive compounds may contribute to improved health outcomes by means of their interaction with the gut microbiota, as illustrated in Figure 2 below. In support of such argument, data accumulated over the last decade points to high fruit and vegetable intakes as factors contributing to improved cognitive function, as well as to a reduced risk of developing neurodegenerative processes such as dementia [144]. Mediterranean-style dietary patterns are also naturally rich in fibre. Both polyphenols and fibre act as prebiotic molecules that will be processed by microbial metabolism [145]. Indeed, the International Scientific Association for Probiotics and Prebiotics (ISAPP) defines a prebiotic as “a substrate that is selectively utilised by host microorganisms conferring a health benefit” and updated that definition in 2017 to acknowledge that “established prebiotics are carbohydrate-based, but other substances such as polyphenols and polyunsaturated fatty acids converted to respective conjugated fatty acids might also fit this definition assuming convincing weight of evidence in the target host” [146].

#### 4.1.2. Prebiotics, More Than Just Fibre

A simpler way to describe prebiotics may be as the non-digestible food ingredients in dietary fibre that nourish and selectively stimulate the growth and/or inhibition of specific colonic bacteria. For example, culinary spices including black pepper, cayenne pepper, cinnamon, ginger, oregano, rosemary and turmeric, to name but a few, have been shown to have prebiotic effects inducing positive changes in human gut microbiota [147]. Another example is provided by proanthocyanidins (PAs), one of the most abundant types of flavonoids in the human diet, which are present in grapes (both in seeds and skins), apples, cocoa, red wine, blueberries, cranberries, bilberries, black currants, hazelnuts, pecans and pistachio nuts. PAs are processed by certain microbial genera, e.g., *Clostridium* and *Eubacterium* [148]. Therefore, clinicians reviewing the results of 16S-type microbial rRNA assays could expect to see higher numbers of these in individuals whose diets regularly feature these foods. This was confirmed recently by the results from a recent open-platform citizen science microbiome research project known as the “American Gut” project [149] that found emergent positive associations among the microbiome, metabolome and the diversity of plant-based foods consumed by the over 10,000 participants. Researchers found that the wider the diversity in fruits and vegetables consumed, the wider the microbial diversity. Additionally, comparing living data across cohorts, the project also confirmed existing associations between the microbiome and psychiatric illness. Could vegetable fibre have contributed to the higher microbial diversity? A review of the literature on that subject seems to point that way [150,151,152]. Therefore practitioners can expect to see an increase in their patients’ microbial diversity as a result of consuming a prebiotic-rich diet. It is worth pointing out that, with the amount of evidence available, it is safe to consider the concept of “polyphenols as antioxidants” as somewhat outdated. Instead, clinicians should consider these compounds as providers of health benefits resulting from microbial metabolism. Therefore, nutrition practitioners should regard dietary polyphenols as contributors to the maintenance of gastrointestinal health “by preserving microbial balance through the stimulation of the growth of beneficial bacteria (i.e., Lactobacilli and Bifidobacteria) and the inhibition of pathogenic bacteria, exerting prebiotic-like effects” [153]. Furthermore, there is evidence that the metabolism of polyphenols by gut microbiota increases their bioavailability to the host [154,155].

#### 4.1.3. Working with Microbial Diversity in Clinical Practice

Nutrition practitioners can manipulate their patients’ microbial diversity and abundance with simple dietary prescriptions. And given the strong indication in current literature that the more varied the diet and its ingredients, the more diverse the gut microbiota is likely to be, it would appear to be appropriate for practitioners to recommend eating a rainbow of brightly-coloured fresh foods that are also good sources of fibre on a daily basis. Cruciferous (e.g., broccoli, cabbage, cauliflower) and dark and green leafy vegetables (e.g., kale, chard) vegetables, as well as bulbs (e.g., garlic, onions, spring onions/shallots, leeks) are amongst those that provide higher amounts of fibre along with nutrients known for their neuroprotective activity, such as folate and sulforaphane [156,157].

Although studies confirming the activity of specific foods on neuroprotection are only starting to emerge, given the safety of the proposed interventions, it would seem pertinent for these foods to feature more prominently in dietary programs designed to improve cognitive function outcomes by means of working on the microbiota-gut-brain axis. Funding for randomised controlled trials to assess the impact of these simple food-based interventions on brain health via the gut is unlikely to become available anytime soon; therefore, more real-world research needs to be documented to help practitioners understand the changes to specific microbial genera that are triggered by these types of safe dietary changes.

### 4.2. Faecal Calprotectin and the Brain

Whilst faecal calprotectin is an inflammatory marker used to assess the presence and severity of inflammatory bowed diseases (IBDs) such as Crohn’s disease (CD) and ulcerative colitis (UC) [158], emerging evidence suggests it could also be useful in the assessment of cognitive decline. A recent study carried out on 22 Alzheimer’s patients by Leblhuber et al. [159] showed that almost three quarters of AD patients presented with faecal calprotectin concentrations higher than normal (>50 mg/kg). This is interpreted to be a sign of intestinal permeability/leaky gut, where faecal calprotectin has translocated from the gut into systemic circulation as a result of a disturbed intestinal barrier function. Calprotectin is a heterodimer formed by pro-inflammatory proteins S100A8 and S100A9 and, incidentally, the latter has been established as a biomarker for the diagnosis and progression of AD and dementia [160].

#### Faecal Calprotectin: Clinical Considerations

Faecal calprotectin tests are readily available to nutrition and lifestyle medicine practitioners. Given the evidence available so far, it would be prudent to take a high reading (typically over 50 mcg/g) into consideration for patients who may present with cognitive impairment, independently of any IBD diagnosis.

Practitioners wishing to act directly on calprotectin levels by means of nutritional interventions may wish to consider the results of a prospective, double-blind, crossover and with placebo study carried out on 30 cystic fibrosis patients from two Spanish hospitals who were administered a chewable tablet containing 10^8^ CFU of *Lactobacillus reuteri* DSM 17938 for six months and who experienced statistically-significant reductions in calprotectin levels compared to controls [161]. Similar outcomes were experienced by children with cystic fibrosis in another controlled trial where those randomly allocated to the intervention group received 6 × 10^9^ CFU of Lactobacillus rhamnosus GG (LGG) once daily for one month. Their faecal calprotectin experienced a significant reduction compared to those in the placebo arm of the study. Additionally, those in the intervention group also experienced the added benefit of improved microbial diversity [162].

### 4.3. Zonulin and Intestinal Permeability

Zonulin is a physiological modulator of intercellular tight junction function and thus a regulator of gut permeability [163,164]. Elevated zonulin levels have been found in coeliac disease [165,166], but high zonulin levels are not always associated with gastrointestinal symptoms. In fact, based on a recent study carried out on the offspring of the participants of the Malmö Diet and Cancer cardiovascular cohort (*n* = 363), Ohlsson et al. identified stronger statistical correlation between high zonulin levels and higher waist circumference, diastolic blood pressure, fasting glucose and increased risk of metabolic diseases [76].

#### 4.3.1. Zonulin and Gluten

Zonulin levels also seem to be raised in patients who seek the advice of nutrition practitioners as they experience reactions to gluten-containing foods that range from mild to severe, but who have tested negative for coeliac disease. Whether it is the gluten itself that it is the culprit in this kind of situations, that is a different matter. Based on real-world evidence, the discomfort experienced by these patients is most likely to be a combination of factors: gluten, polyols, stress and dysbiosis, as well as loss of mucosal barrier integrity.

#### 4.3.2. Zonulin and Potential Clinical Presentations: Non-Coeliac Gluten Sensitivity

A small number of individuals do suffer from a condition that has been termed “non-coeliac gluten sensitivity” (NCGS), where dysbiosis can manifest itself as gastrointestinal inflammation, diarrhoea and/or constipation, visceral hypersensitivity, abdominal pain, a dysfunctional metabolic state characterised by enhanced energy harvest and deranged peripheral immune and neuro-immune communication, a pathologic cascade that may promote oxidative stress, neuroinflammation and cognitive dysfunction [167]. NCGS is a newly-identified pathological entity that describes the symptoms experienced by non-coeliac subjects [168,169]. They experience coeliac-like symptoms as a result of wheat intake, including IBS-like symptoms including abdominal pain, nausea, bloating, flatulence, diarrhoea or constipation [170], with gluten as the confirmed causative factor. Although it is tempting to assume that many of the patients who seek the advice of nutrition practitioners may be suffering from NCGS, in a recent small-scale study by Bardella, Elli et al. [171], only 8% of 37 patients were diagnosed with NCGS. The rest of the participants were affected by fermentable, oligo-, di-, mono-saccharides and polyols (FODMAPS) in wheat, but not by gluten itself. This is an important fact to take into account to avoid misdiagnosis [172], as there is compelling evidence that fructans and galactans in wheat can trigger the same kind of symptoms as gluten does in NCGS [173]. Regardless of the actual trigger for these NCGS symptoms, there is also a subset of patients who experience mostly neurological disturbances as a result of consuming gluten and polyol-containing foods. The symptoms documented as a result of consumption of gluten-containing foods include psychotic-type episodes that can range from mild anxiety to depression or even hallucinations. Catassi [174] and Casella et al. [175] mention how some of these symptoms may be contributed to by a temporary increase of blood anti-gliadin deamidated antibodies (IgG), and Lionetti et al. [176] report the gradual improvement and return to normality a few days after removing gluten from the patients’ diets.

#### 4.3.3. Zonulin and Parasitic Infections

There is evidence that common parasitic infections can be detrimental to the integrity of the mucosal tissue of the gut, contributing to intestinal permeability. Infections with protozoan parasites such as *Blastocystis hominis* and *Giardia intestinalis* have been reported to cause damage to the intestinal wall [177]. *Blastocystis hominis* is mostly non-pathogenic, but can cause IBS-like symptoms [178,179]. Therefore, if stool zonulin levels were seen to be raised in a patient with cognitive impairment, it would be reasonable for practitioners to consider protozoan infections as a potential contributing factor to the impaired barrier function of the intestinal mucosa that zonulin is confirming. At this point, it would be appropriate to treat that infection in order to minimise further damage. The probiotic yeast *Saccharomyces boulardii* has been reported to have similar effectiveness to metronidazole for the treatment of *Blastocystis hominis* infection in children [180], and there is plentiful unreported data from practitioners who have used this natural agent in the treatment in adults presenting with *B. hominis* with a high degree of success. This is another instance where practitioner-researchers could contribute with real-world data on the effectiveness of this natural line of treatment. If a drug must be used, *B. hominis* has been shown to exhibit good sensitivity to metronidazole [181].

#### 4.3.4. Reducing Zonulin Levels

Aside from limiting exposure to the triggers discussed previously in this section, practitioners wishing to reduce zonulin levels in order to improve intestinal barrier integrity may consider supporting the process by adding nutraceuticals with known clinical efficacy and applicability to human health. For example, in a randomised, double-blind, placebo-controlled trial conducted at the Institute of Nutrient Research and Sport Nutrition in Graz, Austria, 52 endurance trained men and women between 20 and 50 years of age and similar in body composition ratios received 1.85 g of zeolite per day for 12 weeks or a placebo. Although the study did not find out exactly how zeolite worked, the individuals who received the zeolite supplementation experienced decreased levels of stool zonulin along with beneficial effects on intestinal wall integrity such as mild anti-inflammatory effects [182]. Similarly, another double-blind placebo-controlled study compared supplementation with 500 mg of colostrum bovinum for 20 days with a placebo (whey) on 16 athletes with intestinal permeability and found that those taking the colostrum experienced a small, but significant decrease in stool zonulin levels compared with control [183]. A further double-centre and double-blind randomized clinical trial carried out by Liu et al. [184] at the Gastrointestinal Institute of Sun Yat-sen University, carried out on 150 patients with colorectal carcinoma undergoing colectomy, found that the 75 patients in the intervention group (who were supplemented daily with 2 g a probiotic mix given orally for six days preoperatively and 10 days post-operatively) experienced a reduced rate of postoperative septicaemia alongside with reduced serum zonulin concentrations compared with controls. The probiotic mix contained *Lactobacillus plantarum* (CGMCC No. 1258, cell count ≥10^11^ colony forming units per gram (CFU)/g), *Lactobacillus acidophilus*-11 (cell count ≥ 7.0 × 10^10^ CFU/g) and *Bifidobacterium longum*-88 (cell count ≥ 5.0 × 10^10^ CFU/g) in an acid-resistant coating at a total daily dose of 2.6 × 10^14^ CFU.

### 4.4. Short Chain Fatty Acids

Acetate, butyrate and propionate are short chain fatty acids (SCFAs) that are produced by certain classes of bacteria [185] amongst which are *Bacteroides*, *Bifidobacterium*, *Clostridium*, *Eubacterium Lactobacillus*, *Prevotella*, *Propionibacterium* and *Roseburia* [186]. The Bacteroidetes phylum is known to be the main producer of acetate and propionate, whilst Firmicutes generate most of the butyrate [187]. Aside from serving predominantly as an energy substrate for colonocytes and enterocytes [188], butyrate also inhibits inflammatory responses through NF-kappaB (NF-κB) inhibition [189]. Propionate is mostly absorbed by the liver and has also been reported to inhibit NF-κB, as well as to improve insulin sensitivity [190], whilst acetate is mostly released into circulation so that it can reach peripheral tissues, including the brain [191,192]. Both propionate and acetate have been found to increase satiety [193,194]. Acetate and butyrate are also structurally related to ketone bodies, acetoacetate and d-β-hydroxybutyrate, respectively, both of which are showing promising effects in neurological disorders [195,196]. Cunnane et al. [197] document how supplementation with ketone-inducing medium chain triglycerides helps achieve a state of moderate ketosis that may stimulate mitochondrial biogenesis whilst improving oxidative phosphorylation and ATP generation in the brain. This type of approach is still experimental, so relying on gut microbes to provide us with good amounts of SCFAs by means of fibre metabolism may be a safer alternative until further clinical data are available. The advantage of relying on colonic microbiota for the production of these acidic metabolites is that SCFAs are not secreted in isolation, but concomitantly with a myriad of other compounds that “lubricate” the neural, neuroendocrine and neuroimmune communication channels between gut microbes, the enteric nervous system and the central nervous system [198].

Aside from its relationship with ketone bodies, butyrate has received additional attention because of its ability to act epigenetically as a deacetylase inhibitor [199]. The pioneering experimental work by Braniste et al. [200] at the Karolinska Institute, where sodium butyrate was administered to mice in order to decrease the permeability of the blood brain barrier (BBB) by increasing the expression of a protein called occludin, a transmembrane protein of the tight junction. Further, Muraca et al. [201] and Ahmadi Badi et al. [202] describe how nanoparticle-sized by-products of bacterial metabolism known as outer membrane vesicles (OMVs) are able to travel through into the bloodstream in situations where the integrity of the intestinal barrier is disrupted, eliciting a low-grade inflammatory response that can also affect the permeability of the BBB. These emerging arguments have sparked discussions about whether the BBB may also be sensitive to changes in the gut microbiota, and whether a potential correlation between a leaky gut and a “leaky brain” exists. Albeit plausible, these hypotheses have not yet been backed up by strong enough evidence from either human or animal studies, and further research is needed to test them. 

#### Clinical Insight on SCFAs

**Figure 3:**
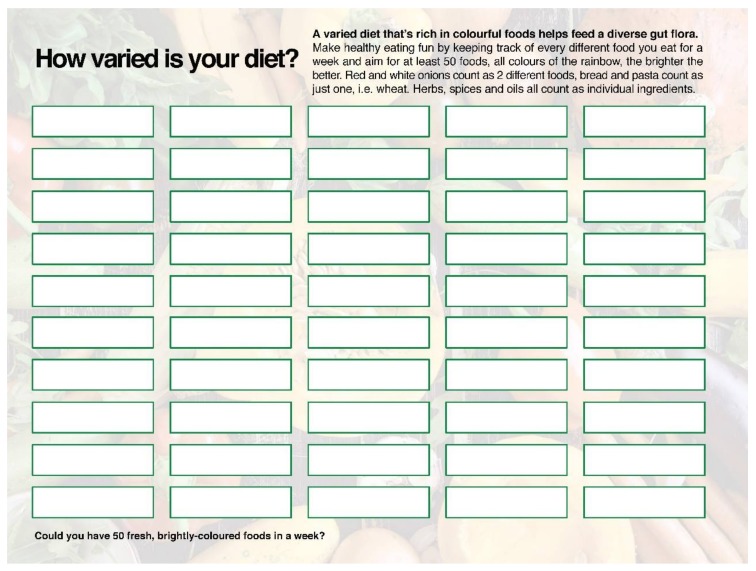
The author’s “50-food challenge” chart is an example of a simple, but powerful data collection tool used in clinical practice to engage with patients in a light-hearted way so that they report back to their practitioner on their dietary diversity. The rationale is to motivate patients to vary the foods they have every day, so that they’re increasing their micronutrient diversity, thereby feeding different classes of gut microbes.

Well-controlled human intervention studies investigating the role of SCFA on cognitive health are eagerly awaited. Until that time, practitioners who identify low levels of SFCAs in the stool test results of a cognitively impaired patient are urged to look at their dietary diversity, and particularly at the sources of fibre. Increasing the diversity of brightly-coloured plant foods, even in small amounts, can have a dramatic effect on SCFA production. As an example, the author uses a “50-food challenge” chart as a clinical tool to raise his patient’s awareness of the diversity (or lack thereof) in their diet over a seven-day period and to make them choose fresh fruits, vegetables, herbs and spices that they would not normally include in their shopping baskets. The aim of this exercise is to provide a wide range of prebiotic substances that feed as many bacterial classes as possible, thereby promoting an increase in SCFA levels in a natural way that is entirely food-based. The “50-food challenge” chart is featured in Figure 3 below.

### 4.5. Beta-Glucuronidase

Glucuronidation is one of the phase II detoxification pathways whereby mammals inactivate toxic compounds by linking them to a glucuronic acid sugar molecule for GI excretion [203]. Certain types of bacteria, including *Clostridia* and *Enterobacteriaceae* [204], express an enzyme called beta (β)-glucuronidase that removes the glucuronic acid as a carbon source, effectively reversing the actions of said inactivation [205].

#### Clinical Notes on Beta-Glucuronidase

β-glucuronidase is also responsible for the deconjugation of oestrogens into their active forms. Therefore, this enzyme has the ability to influence non-ovarian oestrogen levels via enterohepatic circulation [206]. Oestrogen works throughout the entire brain of both males and females and is involved in a variety of brain functions, including memory [207]. In patients presenting with dysbiosis and low microbial diversity, practitioners should look out for low β-glucuronidase levels as these may result in a reduction in circulating oestrogens that could contribute to loss of cognitive function [21]. Conversely, when patients present with high β-glucuronidase levels, practitioners advised to look out for potential inactivation of toxin disposal via decreased glucuronidation. This increases exposure to circulating endobiotic and xenobiotic compounds [208,209] and increases the chances of further dysbiosis taking place as a knock-on effect, mediating further damage to the integrity of the intestinal barrier [210,211].

Glucaric acid modulates the activity of β-glucuronidase and is present naturally in fruits like apples [212] and grapefruits [213], as well as in fermented foods like kombucha [214]. Glucaric acid is also available in nutraceutical form as calcium d-glucarate, which has been used to help lower raised β-glucuronidase levels [215]. The author’s preferred source of glucaric acid is kombucha. The additional advantage lies within the fact that this fermented tea drink also provides a rich source of hepatoprotective enzymes [216] along with a myriad of antibacterial and antifungal polyphenolic molecules [217,218] that can provide great support for an individual suffering from dysbiosis and in need of gut-restorative, functional foods.

## 5. Dietary Neuroprotection, from the Gut Up

With advancing knowledge on the communication system that connects the gut and the brain, clinicians are now able to tackle neuroprotection upstream from the gut. In fact, working on neuroprotection starting in the gut can be a very satisfying job for nutrition and lifestyle medicine practitioners. As outlined above, nutrition interventions consist mainly in the use of prebiotics and probiotics, both of which have been used traditionally to support gastrointestinal health.

### 5.1. Prebiotics, Probiotics and Psychobiotics

Manipulating gut bacteria through the use of prebiotics and probiotics need not be complicated. Despite the lack of precise human clinical data, the author has found that simple tactics such as increasing dietary diversity by using the “50 food challenge” chart or similar can be easily implemented in patients’ busy lifestyles, bringing about a profound positive influence on both gut health and emotional wellbeing that is starting to be confirmed by emerging evidence such as the results of the “American Gut” project mentioned above [149] and its links with improved mental health, as well as other small-scale human experiments that clinicians can use as reference points. For example, Schmidt et al. [219] measured the cortisol levels of 45 participants as a means to assess their emotional processing, whilst taking either a prebiotic fibre supplement or a placebo for three weeks. Participants receiving the prebiotic supplements showed increased attentional vigilance to positive versus negative stimuli similar to those seen following administration of pharmacological agents such as the selective serotonin reuptake inhibitor citalopram or the benzodiazepine diazepam in healthy individuals. This mode of action by live bacteria or probiotics that, when ingested, are able “to confer mental health benefits through interactions with commensal gut bacteria” is what Dinan et al. [220] and Sarkar et al. [221] define as “psychobiotics”.

These results are also interesting in that they describe how certain saccharides such as the fibre inulin (present in Jerusalem artichokes, leeks and onions) and other fructo-oligosaccharides, galactooligosaccharides and polydextrose, all of which have been widely used to improve gastrointestinal outcomes, also appear to also influence distant sites, including improvements in neural and cognitive processes, immune functioning and serum lipid profiles [222]. Sources of prebiotic fibre often provide other interesting nutrients that are useful neuroprotective agents. For example, the pseudo-grain buckwheat has been reported to possess interesting prebiotic activity. In vitro and animal studies suggest that some of its bioactive compounds, such as d-chiro-inositol and the flavonoids rutin and quercetin may be partially responsible for the observed neuroprotective effects [223]. Equally, resistant starch from beans, lentils, green bananas and cooked and cooled potatoes and rice is known to have a positive effect on the gut microbiota, increasing the concentration of short-chain fatty acids such as butyrate, increasing insulin sensitivity and improving cardiovascular and kidney health [224].

### 5.2. Fermented Foods as Natural Sources of Probiotics

Fermented foods, based on both dairy and non-dairy substrates, are well-researched tools that help practitioners modulate their patients’ gut microbiota [225], thereby influencing brain health via the many pathways that connect the GI tract with the CNS. As an example, in a recent randomised, double-blind, placebo-controlled trial involving seventy-five petrochemical workers conducted by nutrition researchers at the University of Tehran, the daily addition of 100g of probiotic yoghurt containing two strains of *Lactobacillus acidophilus* LA5 and *Bifidobacterium lactis* BB12 with a total of min 1 × 10^7^ CFU to the participants’ diet resulted in similar improvements to mental health as supplementation with a multispecies probiotic capsule containing seven probiotic bacteria species, *Lactobacillus casei* 3 × 10^3^, *L. acidophilus* 3 × 10^7^, *L. rhamnosus* 7 × 10^9^, *L. bulgaricus* 5 × 10^8^, *Bifidobacterium breve* 2 × 10^10^, *B. longum* 1 × 10^9^,= and *S. thermophilus* 3 × 10^8^ CFU/g, with 100 mg of fructo-oligosaccharide and lactose as carrier substances. The control group was given a conventional yoghurt containing the starter cultures of *Streptococcus thermophilus* and *Lactobacillus bulgaricus* and experienced no statistically-significant improvement in mental health markers, which included changes to experience of depression, anxiety and stress based on validated scales [226]. The comparable effectiveness of the interventions reported in this study supports the use of live probiotic bacteria from whole foods as opposed to the obligatory use of probiotic supplements. Additional advantages to using a whole food approach including lower cost and improved patient compliance.

In another randomised controlled trial, researchers at the University of Connecticut [227] found that pre-menopausal women who ate just 339 g of yoghurt for nine weeks experienced a reduction in biomarkers of chronic inflammation and endotoxin exposure, including lipopolysaccharide (LPS), LPS binding protein (LBP), IgM endotoxin-core antibody (IgM EndoCAb) and zonulin, compared to those who consumed a non-dairy control food. The yoghurt used in the intervention group happened to be low fat. However, based on the natural low fat content of yoghurt, the author questions whether comparable results would have been achieved using full-fat yoghurt.

Yoghurt eaters may also enjoy kefir, a probiotic drink consisting of dairy milk fermented by kefir grains. Kefir is a complex mixture of bacteria and yeasts or scoby (symbiotic culture of bacteria and yeast) that live in a polysaccharide base. Kefir originates from the Caucasus and Tibet [228] and has become widely available in many countries around the world as a “functional food”. Kefir features a number of bacterial species, including *Lactobacillus paracasei* ssp. *paracasei*, *Lactobacillus acidophilus*, *Lactobacillus delbrueckii* ssp. *bulgaricus*, *Lactobacillus plantarum* and *Lactobacillus kefiranofaciens* as the predominant species [229]. *Lactobacillus kefiri*, *Acetobacter aceti* and *Acetobacter rasens* have also been isolated in milk kefir [230,231], as well as in excess of 23 different yeast species, the predominant ones being *Saccharomyces cerevisiae*, *Saccharomyces unisporus*, *Candida kefyr* and *Kluyveromyces marxianus* ssp. *marxianus* [232,233].

Consuming this probiotic drink daily has been shown to increase secretory IgA in faeces, whilst reducing the expression of pro-inflammatory cytokines in the gastrointestinal tract [234]. As discussed previously, inflammation mediates dysbiosis and the subsequent bacterial translocation that contributes to cognitive decline. On that basis, the benefits of downregulating inflammation in the gut are assumed to bring about downregulation of systemic inflammation. This is perhaps why kefir has become favoured by nutrition practitioners as a source of probiotic microbes in a food matrix that’s symbiotic in nature. Many health properties that have been reported with regular consumption, including immunomodulation, antimicrobial and anticarcinogenic activity, as well as the control of serum glucose and cholesterol and the control of lactose intolerance [235,236,237,238].

Non-dairy varieties of kefir have become available in health food shops. Also, availability of kefir grains means that dairy-sensitive patients can even ferment their own non-dairy milk alternatives at home. However, there is a definite benefit in including dairy products in the diets of patients seeking to enhance neuroprotection. In fact, a recent study published in the American Journal of Clinical Nutrition found that individuals who consumed cheese daily had better circulating levels of the endogenous antioxidant glutathione [239]. Glutathione is used by brain cells to fight free radical formation, and cheese and other fermented dairy products such as yoghurt are good sources of probiotic bacteria [240,241]. Therefore, unless patients express specific concerns about dairy, it is advisable that they include it as part of their daily diets. For vegan patients or those who cannot tolerate dairy at all, either because of extreme lactose intolerance or because of sensitivity to milk proteins, e.g., casein, “milk alternatives” made of soy, coconut, oat or almond can be fermented successfully by kefir grains, with soy being particularly suitable in terms of the consistency achieved [242,243].

“Water kefir” grains are naturally dairy-free and are known to ferment a number of non-dairy substrates, including fruit and vegetables juices, and even plain sugared water. These grains contain a variety of microbial species similar to those in milk kefir grains, including *Lactobacillus casei/paracasei*, *Lactobacillus harbinensis*, *Lactobacillus hilgardii*, *Bifidobacterium psychraerophilum/crudilactis*, *Lactobacillus nagelii*, *Lactobacillus hilgardii*, *Leuconostoc mesenteroides*, *Saccharomyces cerevisiae* and *Dekkera bruxellensis* [244,245]. According to Gulitz et al. [246], up to 57 lactic acid bacteria belonging to those species are able to produce exopolysaccharides from sucrose, which makes water kefir a wide spectrum probiotic drink with potentially distinct sensory characteristics to engage individuals with specific dietary preferences.

Other fermented foods that have become popular with practitioners and patients alike as they provide live microbes in a food matrix include sauerkraut and kimchi. Apart from being touted for a range of health benefits [247], some find these “functional foods” [248] much more appealing than taking probiotic supplements. Whilst some of these foods have been used as part of traditional diets, e.g., sauerkraut is a staple food throughout Eastern Europe, some are slightly more exotic. One such example reported in the literature is fermented papaya, which has been used in humans as a wholefood supplement that elicits significant reductions in urinary 8-OHdG, a guanine by-product of DNA breakdown used as a biomarker of genomic instability [249,250]. Further research on the spectrum of activity of these foods as sources of live microbes and particularly their action on the microbiota-gut-brain axis is welcome.

### 5.3. Assessing Clinical Impact

One of the issues facing nutrition and lifestyle medicine practitioners engaging in neuroprotective interventions mediated by their patient’s microbiome is how to assess the effectiveness of their recommendations. Taking into consideration changes in the biomarkers previously discussed in this paper and monitoring the patient’s change over time is absolutely necessary in order to ascertain whether dietary and lifestyle recommendations have had the desired effect. Practitioners recommending nutrition-based interventions to their patients are likely to be exposed to real-world evidence on a daily basis, but unless their recommendations and their effects are appropriately documented this valuable data will remain locked in their patients’ clinical records. As more human evidence emerges and describes the relationship between the gastrointestinal system and the brain, larger numbers of practitioners will be able support their patients’ goals, thereby improving brain health outcomes by working upstream from the gut.

**Figure 4:**
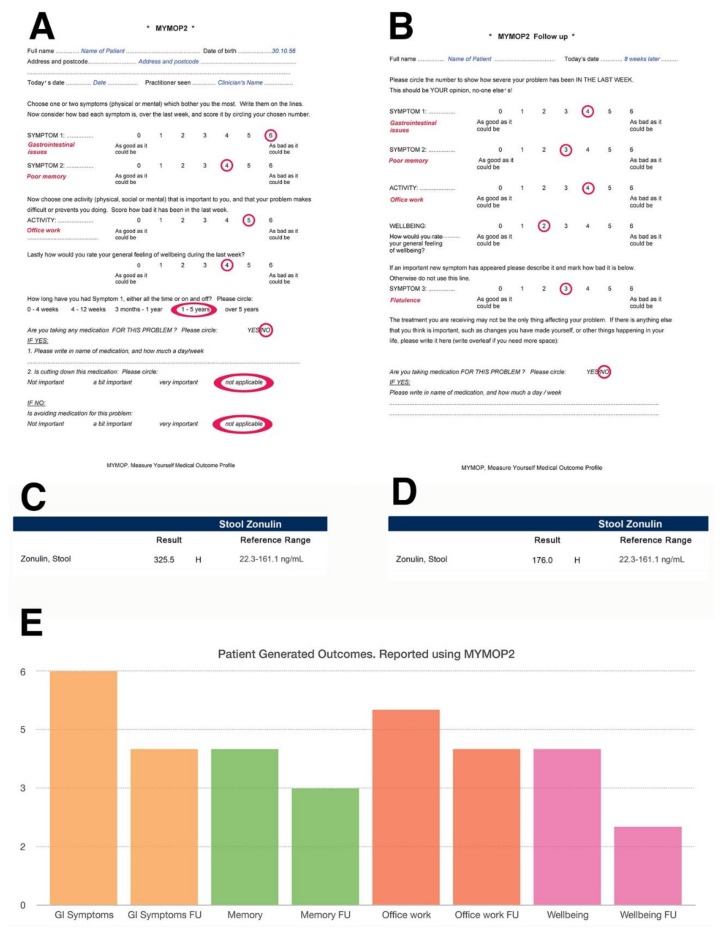
An example of how the MYMOP (Measure Yourself Medical Outcome Profile) questionnaires can be used in clinical practice as a means to assess the effectiveness of interventions. This validated data collection tool is patient-centred and combines qualitative (symptom-based) and quantitative (scoring) data. (**A**) Represents an example of initial MYMOP questionnaire. (**B**) Represents a MYMOP follow-up questionnaire administered eight weeks after the initial one. (**C**,**D**) Represent an example of a marker showing a change as a result to the intervention. (**E**) Represents an example of how data can be organised and displayed using spreadsheet software, e.g., Excel (Microsoft) or Numbers (Apple Inc.), with FU standing for “follow up” in the bar chart.

As an example, Figure 4 illustrates how a practitioner may use a validated tool such as the Measure Yourself Medical Outcome Profile (MYMOP) questionnaire to monitor patient symptoms in relation to their stool zonulin levels over time, using zonulin as a measure of intestinal permeability. Additionally, using a patient-generated outcome tool such as the Measure Yourself Medical Outcome Profile (MYMOP) questionnaire could help practitioners correlate biochemical changes with specific issues affecting the patient, as well as with measures of general wellbeing. The MYMOP questionnaire was created by a general practitioner Dr Charlotte Paterson in the mid-1990s [251] and was then developed and validated by a research team at University of Bristol (U.K.) to be used in any clinical settings where a patient presents with symptoms, which can be physical, emotional or social [252].

Figure 4 illustrates an example where the initial MYMOP is administered and the patient reports “gastrointestinal issues” alongside “poor memory”, stating that these symptoms are of such severity that they really impinge on their ability to work effectively. The MYMOP uses a scale that goes from 0–6 and enables the patient to name the symptoms they wish to tackle at a consultation with their clinician. Zero is the best that symptom can be, whilst six is the worst. Administered eight weeks apart in this example, the MYMOP follow-up questionnaire identifies a slight improvement in symptoms and wellbeing that enables the patient to go about his/her work life more easily. These improvements happen to correlate with a slight decrease in stool zonulin, which may have been brought about by the intervention. The follow up MYMOP also identifies increased flatulence as an additional emerging symptom, which may also provide insight to the practitioner on any side effects of their intervention, i.e., increased fibre from higher vegetable consumption may have triggered the flatulence. The MYMOP tool is validated, takes a couple of minutes to administer, is sensitive to change [253] and has been shown to help with patient engagement [254]. For that reason, it would be desirable for more clinicians to collect patient-generated outcomes using this tool and to share them with other practitioners so that this data is not simply locked away in their patients’ clinical records.

## 6. Discussion

Stool tests provide a holistic snapshot of gut health and microbial diversity, which is not diagnostic in nature, as some of the results could vary from one day to the next. That is why, ideally, a patient should be willing to do a “before and after” test, so that the practitioner has a benchmark for that person against himself or herself prior to the intervention. Based on the author’s own experience as a clinician, patient compliance seems to increase as a result of the process of testing one’s self, i.e., patients seem to be motivated by being shown a test result. Could this be because of a change in expectations by 21st Century patients who seek the support of a practitioner with a whole-person approach? Perhaps this type of patient looks for individualized advice that moves away from the concept of “the average person having an average day” promoted by large-scale trials. These patients expect their clinician to take personalisation to the next level making care participative by involving them in the process, accounting for differences not only in their gut microbes and metabolites, but in their environment and their lifestyle. This more demanding patient does not want their clinician to wait for years so that science can make it from bench to bedside and are happy to engage in “one-person trials”, described by Schork [255] as the basis for personalised medicine. The author is of the view that practitioners should see every patient as a research subject, every single meal as an opportunity for treatment and every food as a potential drug [256]. However, when working with a complex environment such as the gut, absolute precision is not realistic, so both patients and clinicians need to understand that many of these interventions, whilst safe in principle, involve a certain amount of trial and error that future good quality research should help minimise.

Research into the modulation of the gut-brain axis via the gastrointestinal microbiota is still an emerging, frontier science. A large proportion of the evidence available is based on either basic science and animal models that may lack translatability into measurably effective human interventions. Therefore, highly sophisticated, individualized prescriptions of specific prebiotic compounds and probiotic strains that would constitute the ideal of personalization for nutrition and lifestyle medicine practitioners still remains somewhat utopian. The reality is that simpler dietary interventions can be extremely powerful as well. As an example, it has been highlighted how prescribing an increased diversity of brightly-coloured fruit and vegetables that are rotated weekly in order to provide a varied source of substrates for the microbiota to feed on and metabolise therapeutic metabolites can have positive effects. Moderate levels of physical activity, as well as the use of stress management techniques, e.g., breathing exercises, also pose minimum risk of harm to the patient compared with the potential benefit. Practitioners are advised to use their clinical judgement and weigh the likely benefits against any potential disadvantages, whilst always taking into consideration their patient’s values and preferences, as well as the best available scientific research to support their clinical decisions. While we wait for good quality RCTs to be published, food-based interventions are likely to be extremely safe for most (e.g., “eat a wider variety of vegetables instead of always eating the same 2 or 3 every day of the week”), so practitioners could be a little adventurous in the clinical application of emerging findings. Ongoing assessment of and patient outcomes derived by these interventions is needed in order to minimise risk whilst potentially maximising health benefit.

Last, but not least, this paper identifies an urgent need for more nutrition and lifestyle medicine practitioners to adopt the role of practice-based researchers who can help articulate real-world evidence emerging from real cases they deal with in their own clinical practice. Indeed, the author urges the growing number of practitioners using stool tests to assess their patients’ health to take the role of researchers in their own practices and to document the findings of their “experiments” as real-world evidence that otherwise remains locked away in their patient records and could inform the clinical decisions of peers managing the same type of issues. This could be as simple as collecting self-reported patient-generated outcomes as measures of effectiveness. Sharing the results of their *n* = 1 interventions with other practitioners using similar assessment tools is likely to enable practice-based learning and ongoing refinement of clinical applications of scientific findings. However, both clinicians and patients need to be mindful of the complexity of the microbial ecosystem and the fact that host-microbe interaction is equally complex. The tools discussed in this paper provide useful answers to clinical questions, but cannot be seen as “one-fits-all” solutions in addressing patients’ health.
